# Heat Stress and Non-Traditional Chronic Kidney Disease (CKDnt) Among Nepali Migrant Workers: A Climate-Sensitive Global Health Perspective

**DOI:** 10.7759/cureus.108607

**Published:** 2026-05-10

**Authors:** Roshan Thapa, Sachita Shrestha, Dinesh Neupane, Janavi Wagle, Pinky Jha

**Affiliations:** 1 Internal Medicine, Medical College of Wisconsin, Wauwatosa, USA; 2 Internal Medicine, University of Michigan, Michigan, USA; 3 Public Health Sciences, Johns Hopkins Health System, Baltimore, USA; 4 Internal Medicine, Brookfield East High School, Brookfield, USA

**Keywords:** ckdnt, climate change, global health, heat stress nephropathy, migrant workers, occupational hazards

## Abstract

Climate change-driven heat exposure is an emerging global health crisis with profound implications for outdoor and manual laborers. Among the most concerning outcomes is chronic kidney disease of non-traditional origin (CKDnt), increasingly documented in regions experiencing extreme temperatures. Nepali migrant workers, primarily employed in construction, manufacturing, and agriculture across Gulf Cooperation Council (GCC) countries, face severe occupational heat exposure with limited labor protections. This perspective examines how chronic heat stress contributes to kidney injury among Nepali migrant workers, synthesizing pathophysiologic mechanisms, epidemiologic trends, qualitative evidence on working conditions, and weaknesses in global and national policy responses. Drawing from comparative findings from countries that are global hotspots of CKDnt, we argue that CKDnt reflects a convergence of climate change, labor exploitation, and global inequity. Strengthening Wet Bulb Globe Temperature (WBGT)-based heat-protection protocols, embedding comprehensive renal screening into migration systems, reforming bilateral labor agreements, and advancing international recognition of CKDnt as a climate-sensitive occupational disease are urgent priorities. Addressing this issue is essential not only for safeguarding migrant health but also for advancing climate justice and sustainable development.

## Introduction and background

Extreme heat events, characterized by stagnant warm air masses and persistently high nighttime temperatures, are becoming more frequent, intense, and prolonged worldwide due to climate change, posing substantial occupational health risks for workers engaged in physically demanding labor. The International Labour Organization estimates that more than 2.4 billion workers, over 70% of the global workforce, are exposed to excessive workplace heat, particularly in agriculture, construction, and manufacturing [1]. Extreme heat causes about 22.87 million workplace injuries each year, leading to roughly 18,970 deaths and 2.09 million disability-adjusted life years lost annually [1]. Beyond these acute impacts, excessive heat stress at work is also linked to chronic diseases, including chronic kidney disease of non-traditional origin (CKDnT), a progressive renal condition not attributable to hypertension, diabetes, or other common etiologies [2,3].

An estimated 26.2 million people worldwide live with chronic kidney disease associated with occupational heat exposure [4]. CKDnT was first widely recognized among sugarcane workers in Central America, often referred to as Mesoamerican nephropathy, and has since been reported in South Asia, the Middle East, and parts of Africa [3]. Its geographic distribution closely aligns with regions characterized by extreme heat exposure and strenuous manual labor, supporting a strong occupational and environmental link.

In Nepal, CKDnT is an increasing public health burden and has become an area of concern as the country experiences rising extreme heat events, with temperatures increasing at approximately 0.056°C per year, about twice the global average, with heat-related deaths among adults over 65 more than doubling between 1990-99 and 2014-2023 [5].

Nepali migrant workers represent a particularly vulnerable population within this growing heat-related kidney disease burden. Economic disparities and limited domestic employment opportunities have made Nepal the second-largest labor-exporting South Asian country relative to its population, with remittances contributing nearly one quarter of its gross domestic product [6]. Around 67% of migrant workers are employed in Gulf Cooperation Council (GCC) countries, primarily in construction and infrastructure jobs [7]. These workers, predominantly young men from rural areas, are exposed to extreme heat conditions often exceeding 40-50°C, frequently with limited access to hydration, rest breaks, or health care [8].

During 2009-2017, more than 1,300 deaths among Nepali migrant workers in Qatar were reported, with a pronounced seasonal pattern and higher cardiovascular mortality during hotter periods, consistent with occupational heat stress [9]. Emerging epidemiologic and qualitative evidence indicate increasing reports of renal dysfunction among Nepali returnee workers, many of whom lack traditional risk factors, suggesting CKDnT as a growing occupational health concern in this population [10]. Despite their substantial economic contributions, migrant workers remain highly vulnerable to occupational hazards, and their health outcomes are inadequately monitored.

This review synthesizes current evidence linking occupational heat exposure and kidney disease, with a focus on Nepali migrant workers. By integrating epidemiologic trends, biologic mechanisms, and policy gaps, we situate CKDnT within the broader context of climate change, labor migration, and global health inequities. This paper argues that an effective response requires implementation of evidence-based Wet Bulb Globe Temperature (WBGT)-driven occupational heat protection protocols, combined with mandatory renal surveillance across the migration lifecycle, supported by coordinated global health initiatives and a climate justice framework.

Methods

This study used a narrative review approach to synthesize evidence on occupational heat stress and chronic kidney disease of non-traditional origin (CKDnT) among Nepali migrant workers in Gulf Cooperation Council (GCC) countries. We performed a structured but non-exhaustive search of PubMed, Scopus, and Google Scholar for English-language publications through 2025, using keywords related to heat exposure, CKDnT, migrant labor, Nepal, and Gulf countries cooperation, supplemented by citation tracking.

We included peer-reviewed articles, systematic reviews, and relevant technical and policy reports. Data were thematically synthesized across epidemiology, pathophysiology, and occupational vulnerabilities. Due to heterogeneity and limited Nepal-specific longitudinal data, no meta-analysis was performed.

Global epidemiology

Over the past two decades, chronic kidney disease of non-traditional origin (CKDnT) has emerged as a significant occupational and environmental health concern across multiple regions. Unlike conventional chronic kidney disease, CKDnT predominantly affects young and middle-aged workers without traditional risk factors such as diabetes or hypertension and is strongly associated with prolonged heat exposure and physically demanding labor [2,3].

The epidemic was first recognized in Central America in the 1990s, where disproportionately high rates of kidney disease were documented among male agricultural workers in El Salvador and Nicaragua, particularly in sugarcane-growing regions [11]. Epidemiologic studies reported CKD prevalence exceeding 15-20% in some worker populations, many of whom lacked traditional risk factors [12,13]. Subsequent investigations demonstrated strong associations between occupational heat stress, strenuous labor, and recurrent dehydration. Longitudinal studies further showed measurable declines in kidney function over a single harvest season, supporting a causal pathway linking repeated heat stress to kidney injury [13,14]. Based on accumulating regional evidence, the Pan American Health Organization formalized the term “CKDnT” to describe this condition [15].

Similar patterns have been observed in South Asia, particularly in Sri Lanka and India, where agricultural communities exposed to high temperatures demonstrate increased prevalence of CKDnT [16]. While environmental contributors such as heavy metals and water quality have been proposed, current evidence suggests these factors may be context-specific, whereas heat stress and recurrent dehydration are more consistently observed across affected regions.

The risk of CKDnT also extends beyond agricultural settings. Occupational cohort studies in Thailand have reported elevated kidney disease risk among industrial workers exposed to high workplace heat [17]. In addition, epidemiologic data demonstrate a dose-response relationship between Wet Bulb Globe Temperature (WBGT) exposure and kidney dysfunction among exposed workers, reinforcing heat stress as a key mechanistic driver [18,19]. These findings strengthen the evidence that heat exposure is a central and generalizable occupational risk factor across diverse industries.

Emerging evidence from the Middle East and Gulf countries suggests that migrant workers engaged in construction and infrastructure projects may face similar risks. Occupational studies have documented significant heat strain among workers exposed to prolonged outdoor labor during peak summer months, with WBGT levels frequently exceeding recommended occupational safety thresholds [8]. Large migrant worker cohorts further indicate clinically significant dehydration, heat strain, and inadequate occupational protection in this region [10]. However, compared to Central America and South Asia, data from the Gulf region remain limited, highlighting an important gap in surveillance and research.

Collectively, these findings underscore that climate-related heat exposure is a growing global occupational hazard, disproportionately affecting manual laborers in low- and middle-income regions [4]. Despite increasing recognition, surveillance systems for CKDnT remain underdeveloped, particularly among migrant worker populations, resulting in likely underestimation of the true disease burden.

Pathophysiology

The mechanisms linking occupational heat exposure to chronic kidney disease of non-traditional origin (CKDnT) are complex and multifactorial. Current evidence suggests that CKDnT arises not from a single exposure but from cumulative physiological stress driven by recurrent dehydration, sustained physical exertion, and repeated episodes of subclinical kidney injury in high-temperature environments. Over time, these repeated insults contribute to progressive structural and functional renal damage [2,3].

A central mechanism involves heat-induced reductions in renal perfusion. During exposure to high ambient temperatures, blood flow is redistributed from the core to the skin to facilitate heat dissipation. This thermoregulatory response, combined with plasma volume contraction due to dehydration, reduces renal blood flow [2]. The renal outer medulla, which normally operates under relatively hypoxic conditions, is particularly vulnerable to further reductions in oxygen delivery, increasing the risk of ischemic tubular injury [3].

Dehydration also promotes hyperosmolarity and activation of vasopressin-mediated pathways that may exacerbate renal injury. Experimental and translational evidence suggests that vasopressin activation stimulates the polyol-fructokinase pathway, leading to endogenous fructose production within the kidney. Fructose metabolism subsequently generates uric acid and reactive oxygen species, contributing to oxidative stress, inflammation, and tubular injury [2].

Occupational studies indicate that workers exposed to extreme heat frequently experience recurrent episodes of acute kidney injury (AKI), often subclinical and unrecognized. These episodes may occur repeatedly during work shifts and partially resolve between exposures; however, accumulating evidence suggests that recurrent AKI contributes to long-term kidney damage and progressive decline in renal function [13].

In addition, epidemiologic evidence indicates that higher environmental heat stress, measured using Wet Bulb Globe Temperature (WBGT), is associated with increased risk of acute kidney injury and physiological strain among outdoor workers, supporting a dose-response relationship between heat exposure and renal stress [17,18]. Over time, repeated injury may lead to progressive tubulointerstitial fibrosis, a hallmark pathological feature of CKDnT [16].

Additional contributors may amplify this risk. Exertional rhabdomyolysis under extreme heat conditions can release myoglobin, leading to direct nephrotoxicity and AKI. Concurrent use of non-steroidal anti-inflammatory drugs (NSAIDs) may impair renal autoregulation, particularly in dehydrated individuals, increasing susceptibility to ischemic injury [3].

Environmental heat exposure is commonly assessed using the Wet Bulb Globe Temperature (WBGT) index, a composite measure incorporating temperature, humidity, radiant heat, and wind speed [19]. WBGT levels exceeding 28-30°C are consistently associated with increased heat strain, dehydration, and renal stress in occupational settings [4,18].

Overall, CKDnT appears to develop through a progressive pathway linking recurrent heat stress, repeated acute kidney injury, and chronic tubulointerstitial damage, ultimately leading to irreversible renal disease in vulnerable worker populations.

## Review

Occupational heat exposure among migrant workers

Migrant laborers face occupational and structural vulnerabilities that increase the health risks of extreme heat exposure. Workers in construction, agriculture, and outdoor industrial sectors often perform strenuous labor for prolonged periods with limited access to rest, hydration, or cooling, increasing the risk of dehydration and heat strain. Global assessments by international agencies highlight that rising temperatures are significantly affecting worker health and productivity worldwide [19]. Heat exposure is associated with multiple adverse outcomes, including heatstroke, dehydration, kidney dysfunction, and neurological impairment [19].

In Gulf Cooperation Council (GCC) countries, where summer temperatures frequently exceed 45°C, studies among migrant construction workers have documented clinically significant heat strain, dehydration, and electrolyte imbalance despite seasonal midday work bans [20]. For example, Qatar and the United Arab Emirates implement midday work restrictions during peak summer months; however, enforcement remains inconsistent, with frequent exemptions for “urgent work” [8,20].

Many Nepali migrants work in construction and infrastructure sectors in GCC countries, where prolonged outdoor labor under extreme temperatures increases the risk of heat-related illness and kidney injury. However, Nepal-specific quantitative data on heat exposure (e.g., WBGT exposure profiles and duration of exposure) remain limited, representing a critical research gap. These risks are further compounded by limited healthcare access, restrictive labor systems such as the kafala sponsorship model, and the absence of routine renal screening, allowing CKDnT to remain undetected until advanced stages [10].

Epidemiologic evidence from Nepal

The vulnerability of Nepali migrant workers arises from the convergence of extreme climatic conditions and structurally hazardous labor environments. A growing body of evidence indicates a disproportionate burden of kidney disease among returnee migrants.

A 2025 cross-sectional survey of 404 male patients at the National Kidney Center in Kathmandu reported that one in three dialysis patients had a history of labor migration [21]. Migrant workers initiated dialysis at a markedly younger median age (37 years) compared to non-migrants (54 years) [10,21]. Disease progression also appeared rapid, with many patients being diagnosed abroad and requiring dialysis shortly after returning [10,21].

Importantly, these findings challenge traditional risk factor models. Migrant workers had a substantially lower prevalence of diabetes than non-migrants (10% vs. 34%), and a significant proportion presented without either diabetes or hypertension [10]. This pattern supports an occupational and environmental etiology consistent with CKDnT, indicating that conventional screening strategies focusing solely on metabolic risk factors may miss a large proportion of at-risk individuals.

These observations are supported by earlier studies. A 2023 community-based survey of 718 returnee migrants reported a higher prevalence of reduced kidney function among migrants compared to non-migrants [22]. Additionally, a national survey of nephrologists in Nepal found that the majority of kidney disease cases among returnees were classified as having “unknown etiology,” often lacking traditional comorbidities [23].

Across studies, workers consistently reported prolonged occupational heat exposure, excessive workloads, inadequate rest breaks, and high physical exhaustion. In a cohort of 217 migrant returnees who underwent kidney transplantation between 2013 and 2023, most reported working in temperatures exceeding 40°C, long working hours, low fluid intake, and frequent suppression of urination during work shifts. Additional exposures to dust, chemicals, and aerosols further contributed to high-risk occupational environments [24].

While environmental nephrotoxins (e.g., heavy metals, silica, pesticides) and infections cannot be excluded, the consistent association with occupational heat exposure, combined with the absence of traditional risk factors across multiple studies, supports heat stress as a primary driver of kidney injury in this population [2-4].

Qualitative evidence

Qualitative evidence from returnee Nepali migrant workers and migration health reports highlights consistent links between occupational heat exposure, dehydration, and kidney injury, with workers describing prolonged labor in extreme heat, restricted hydration, and limited access to healthcare [6,12,25]. The following are synthesized themes.

Workplace Environment

Workers described 10-16-hour shifts performing physically demanding labor in extreme heat with minimal shade or rest.

Hydration Barriers

Limited access to water and toilets led to deliberate fluid restriction (“voluntary dehydration”) to maintain productivity and avoid wage loss [12,25].

Lack of Accountability

Workers collapsing from heat illness often faced termination without medical care or compensation. Use of NSAIDs (e.g., ibuprofen, diclofenac) to maintain productivity was commonly reported, despite known nephrotoxic risks [3].

Barriers to Healthcare

Fear of deportation, job loss, language barriers, and high healthcare costs discouraged early care-seeking [12,25].

Lack of Follow-Up Care

Upon return to Nepal, many workers lacked structured medical follow-up or continuity of care [10].

Psychological Distress

Workers reported anxiety, stigma, and financial stress associated with illness and loss of employment [6,12,25].

These qualitative findings help explain quantitative observations: the finding that half of migrant workers initiate dialysis within one year of returning to Nepal [10] becomes more comprehensible in the context of delayed care, workplace termination, and lack of early intervention. Similar patterns have been reported among Filipino and Bangladeshi migrant workers in the GCC, suggesting that this is a broader structural phenomenon rather than one unique to Nepali migrants [12].

Structural determinants

Nepal’s heavy reliance on remittances, which account for approximately one-quarter of its GDP, has created a structural “vicious cycle” in which migrant worker protection is often subordinated to macroeconomic stability [5]. Climate change acts as a critical threat multiplier, accelerating rural livelihood insecurity and increasing economically driven labor migration. Within this “remittance dependency trap,” there is limited political and regulatory incentive to enforce stringent occupational protections or negotiate stronger safety standards with destination countries due to concerns over foreign currency inflows [5,12].

As a result, regulatory gaps, including the absence of mandatory renal health screening, the lack of WBGT-based heat standards in bilateral labor agreements, and insufficient post-return health surveillance, expose migrant workers to hazardous high-heat environments [10,22]. These conditions, combined with exploitative labor practices, increase vulnerability to chronic kidney disease of non-traditional origin (CKDnT), effectively transforming labor migration into a structural occupational health and human rights issue.

For Nepal, the significance of CKDnT in migrant workers is amplified by equity and development considerations. When migrant workers are diagnosed with end-stage renal disease (ESRD), the clinical crisis rapidly evolves into a socioeconomic catastrophe. As young primary income earners, these individuals are typically forced to terminate overseas employment, resulting in immediate loss of household income.

Return to Nepal is accompanied by high and recurrent costs of renal replacement therapy, alongside limited geographic availability of dialysis services. Monthly hemodialysis costs in Nepal are estimated at NPR 15,000-25,000 (USD 110-185), often exceeding or approaching average rural household income (~NPR 12,000) [26]. This mismatch forces families to relocate to urban centers, incurring additional housing and transportation costs, thereby deepening financial precarity and reinforcing cycles of poverty [10,22].

Loss of employment, treatment dependency, and geographic barriers collectively create long-term household vulnerability and intergenerational economic strain [12,22].

Addressing this cascade requires structural reform, including economic diversification beyond remittance dependence and incorporation of enforceable occupational health protections within bilateral labor agreements.

Occupational heat protection policies

Occupational heat protection policies for migrant workers are primarily implemented in destination countries, particularly within the Gulf Cooperation Council (GCC) region, including Qatar, the United Arab Emirates, Oman, and Saudi Arabia (Table 1). While these countries have introduced formal heat mitigation measures, such as midday work bans during peak summer months and, in selected jurisdictions, the application of Wet Bulb Globe Temperature (WBGT)-informed thresholds, implementation remains uneven across sectors. Enforcement challenges are particularly evident in construction and subcontracted labour environments, where migrant workers are disproportionately represented [26]. As a result, the effectiveness of these policies is constrained by variability in compliance monitoring and limited occupational health surveillance systems [8,19].

**Table 1:** Existing policies in common destination countries for migrant workers

Destination Country Policy	Type of Protection	Critique
Qatar (2021)	WBGT-triggered stop-work thresholds; Worker’s right to stop work (Government of Qatar, 2021) [27]	While science-based, implementation is inconsistent, and thresholds are arguably still too high for strenuous labor.
UAE (2024)	Midday break (12:30–3:00 PM); calendar-based (Ministry of Human Resources & Emiratisation, 2024) [28]	Time-based bans fail to address high WBGT levels occurring outside mandated hours or months, rendering them medically inadequate. Also, Urgent nature work made it frequent to violate these break
Oman (2025)	"Safe Summer" campaign; non-binding guidelines (Oman Ministry of Labour, 2025) [29]	Reliance on campaigns and non-binding guidelines lacks enforceability and employer accountability.

In contrast, Nepal’s occupational health and migration governance framework demonstrates significant structural gaps. Pre-departure health assessments remain largely focused on communicable diseases and general fitness evaluations, with limited integration of occupational exposure risk screening, including heat-related kidney injury [10,22]. Furthermore, Nepal lacks a systematic post-return health surveillance framework, particularly for CKDnT, resulting in missed opportunities for early diagnosis and intervention. The Labour Act 2017 provides a general legal foundation for worker protection; however, it does not explicitly address occupational heat stress monitoring or long-term renal health risks in migrant populations [22].

Knowledge gaps and research priorities

Despite growing recognition of CKDnT among migrant labor populations, several critical evidence gaps remain, particularly in relation to Nepali migrant workers in Gulf Cooperation Council (GCC) countries.

First, there is a fundamental lack of baseline renal function data prior to migration. No studies have established pre-departure measures such as estimated glomerular filtration rate (eGFR) or albuminuria among Nepali workers. As a result, it remains impossible to distinguish pre-existing chronic kidney disease from kidney injury acquired during occupational exposure abroad, limiting causal inference.

Second, there is a complete absence of longitudinal follow-up studies among returnee workers. Existing evidence is predominantly cross-sectional, capturing kidney dysfunction at a single time point. This leaves a major gap in understanding disease progression after return, including decline trajectories and modifiable risk factors.

Third, occupational heat exposure has not been objectively quantified in this population. No studies have measured environmental heat stress using standardized indices such as Wet Bulb Globe Temperature (WBGT), nor physiological strain markers such as core temperature or hydration biomarkers during actual work exposure in GCC settings [19].

Fourth, there is a paucity of qualitative research exploring Nepali migrant workers’ lived experiences. Unlike evidence available from other regions such as Mesoamerica, there is limited context-specific qualitative data on heat exposure perceptions, occupational health behaviors, or barriers to healthcare access among Nepali workers [12,25].

Fifth, the economic burden of CKD in Nepali migrant households remains unquantified. There is a lack of data on direct and indirect costs, including dialysis expenses, catastrophic health expenditures, loss of income, and long-term household impoverishment following return migration and disability [25,30].

Finally, there is a complete absence of intervention-based research in this population. No studies have evaluated preventive strategies such as WBGT-informed work-rest cycles, hydration protocols, or structured renal screening programs for early detection of CKD risk among Nepali migrant workers.

Collectively, these gaps highlight the urgent need for prospective cohort studies, occupational exposure assessments, qualitative inquiry, economic evaluations, and intervention trials tailored specifically to Nepali migrant populations.

## Conclusions

CKDnT among Nepali migrant workers is a climate-sensitive occupational health crisis driven by extreme heat exposure, dehydration, and structurally unsafe labor conditions. Despite the growing burden, the true prevalence of CKDnT in Nepal remains unknown due to the absence of a comprehensive nationwide surveillance and screening system.

At the same time, Nepal lacks specific, enforceable policies to protect migrant workers both domestically and abroad in GCC countries. This crisis demands urgent action aligned with the WHO and ILO occupational heat safety principles. Priorities include WBGT-based work-rest standards, mandatory renal screening before migration and after return, continuous occupational health surveillance, and formal recognition of CKDnT as a climate-related occupational disease.

Equally critical are enforceable workplace protections ensuring access to hydration, shade, rest breaks, and the right to refuse unsafe work without retaliation. Without immediate structural reform, including strengthened bilateral labor agreements and enforceable occupational protections, rising global temperatures will accelerate preventable kidney failure in already vulnerable migrant populations. This represents a pressing issue of climate justice, occupational safety, and global health equity requiring an urgent coordinated international response.
